# SOCIAL SUPPORT, SOCIAL STRAIN, AND DENTAL CARE UTILIZATION AMONG OLDER CHINESE AMERICANS

**DOI:** 10.1093/geroni/igac059.1101

**Published:** 2022-12-20

**Authors:** Weiyu Mao, Bei Wu, Iris Chi, Wei Yang, XinQi Dong

**Affiliations:** University of Nevada, Reno, Reno, Nevada, United States; New York University, New York, New York, United States; University of Southern California, Los Angeles, California, United States; University of Nevada Reno, Reno, Nevada, United States; Rutgers, New Brunswick, New Jersey, United States

## Abstract

Regular dental care utilization is instrumental to good oral health. This study aimed to examine how positive and negative aspects of social relationships jointly exert influences towards dental care use among foreign-born older Chinese Americans. Data came from the Population Study of Chinese Elderly in Chicago collected between 2017 and 2019 (n = 3,000). Dental care utilization was dichotomized into “no dental visit” versus “any dental visit” in the past two years (including dental visit overseas). Social support and strain were measured by the Health and Retirement Study’s scale from spouse, other family members, and friends (1= having any support/no strain). In stepwise logistic regression, accounting for chronic conditions, oral health problems, and sociodemographics, spousal support remained to be significantly associated with a lower likelihood of having any dental visit. Findings illustrate the importance of understanding how different aspects of social relationships might play a role in dental care use.

